# A Medical Service for All

**Published:** 1920-12-11

**Authors:** 


### A MEDICAL SERVICE FOR ALL.

Since the publication of our last number there has appeared the interim report of the Consultative Council on Medical and Allied Services appointed by the Scottish Board of Health—and the ideas contained in this anxiously awaited document may have done much to provide a working basis for our medical reconstruction. The companion, but earlier, English report, to which we referred last week, was undoubtedly open to criticism, in that it expressed only the ultimate ideal and gave little indication of how we should set about attaining it. Also, if viewed as a scheme immediately to be brought into operation, it would be uncommonly expensive. But in this latest suggestion there are simpler ideas expressed and an obvious realisation of the time which full development of a national health service will inevitably take.

At the close of last week the daily press provided excellent summaries of this report, and it is unnecessary either to repeat their *précis* or to express their agreement that a complete and adequate medical service should be brought within easy reach of every member of the community. The report's most important statement is, however, the recommendation that this service should not *all* be provided at the public expense, and that it should not be furnished to everyone by a body of State or civic officials and practitioners. The State may and should take an active part in helping to bring about the necessary organisation, but the direct State medical service principle, as we know it, should be applied only to those people who come under the national insurance grade.

Now, the recent Ministry of Health activities in appointing regional officers and attempting to extend the insurance practice efficiency are part and parcel of the last scheme, and, without discussing its value in that respect, we can dismiss it as the second part of the recommendations already in process of fulfilment.

On the other hand, the remaining half of the report appears to carry with it a direct exhortation to the medical profession to organise *itself* to deal with that section of the population above the insurance practice standard of income, and to carry out this organisation under the guidance and authority of one of its own recognised associations or representative professional bodies. It is apparently suggested that a health service should be formed based upon the family, not the person, as the normal unit, and with the family doctor as the normal medical attendant. The resources of all available local health agencies should be made readily applicable to the patient, but through the medium of his family doctor, and the question of local "consultants" might well be solved by genuine professional selection—a local vote possibly—among the doctors of the district. In other words, it is urged that the doctors, under their own professional authorities' guidance, should proceed to fit themselves under an adequate system, the older methods of isolated and throat-cutting practice being as much as possible submerged in a national service, not financed by the State, but on the principles of family practice and where the first key to advancement shall be true professional excellence.

Again, it is something of an idealisation, but there seems no reason why it should not gradually materialise, if by adopting this plan the Government could openly invite the medical profession to put its own house in order and run its own service. If taken up, it then remains for the controlling and representative medical bodies to set at once about their task. If success were achieved, the benefit to the profession as a whole would be unquestionable, and in the building of such a scheme might develop some degree of that urgently needed professional unity, which of late has apparently receded more and more into the realms of improbability. There seems to us to be a great work ahead for the new Federation, if the doctors will hold together and keep the true ideals of a noble profession before them.

